# BDNF Therapeutic Mechanisms in Neuropsychiatric Disorders

**DOI:** 10.3390/ijms23158417

**Published:** 2022-07-29

**Authors:** Amjad H. Bazzari, Firas H. Bazzari

**Affiliations:** 1 Faculty of Medicine, Arab American University, 13 Zababdeh, Jenin 240, Palestine; 2 Faculty of Pharmacy, Arab American University, 13 Zababdeh, Jenin 240, Palestine; firas.bazzari@aaup.edu

**Keywords:** brain-derived neurotrophic factor, TrkB signaling, neuroprotection, synaptic plasticity, neuromodulation, neurodegeneration, neuroinflammation, oxidative stress

## Abstract

Brain-derived neurotrophic factor (BDNF) is the most abundant neurotrophin in the adult brain and functions as both a primary neurotrophic signal and a neuromodulator. It serves essential roles in neuronal development, maintenance, transmission, and plasticity, thereby influencing aging, cognition, and behavior. Accumulating evidence associates reduced central and peripheral BDNF levels with various neuropsychiatric disorders, supporting its potential utilization as a biomarker of central pathologies. Subsequently, extensive research has been conducted to evaluate restoring, or otherwise augmenting, BDNF transmission as a potential therapeutic approach. Promising results were indeed observed for genetic BDNF upregulation or exogenous administration using a multitude of murine models of neurological and psychiatric diseases. However, varying mechanisms have been proposed to underlie the observed therapeutic effects, and many findings indicate the engagement of disease-specific and other non-specific mechanisms. This is because BDNF essentially affects all aspects of neuronal cellular function through tropomyosin receptor kinase B (TrkB) receptor signaling, the disruptions of which vary between brain regions across different pathologies leading to diversified consequences on cognition and behavior. Herein, we review the neurophysiology of BDNF transmission and signaling and classify the converging and diverging molecular mechanisms underlying its therapeutic potentials in neuropsychiatric disorders. These include neuroprotection, synaptic maintenance, immunomodulation, plasticity facilitation, secondary neuromodulation, and preservation of neurovascular unit integrity and cellular viability. Lastly, we discuss several findings suggesting BDNF as a common mediator of the therapeutic actions of centrally acting pharmacological agents used in the treatment of neurological and psychiatric illness.

## 1. Introduction

Various neurotrophic peptides are expressed across the central nervous system (CNS) and primarily function as viability signals. During development, neurotrophins promote neurogenesis in addition to neural differentiation and maturation [1]. In the adult brain, neurotrophins serve to maintain the function and survival of neurons [2]. The most abundant among these is brain-derived neurotrophic factor (BDNF), which is expressed in many organs and by neurons and glial cells, especially astrocytes, throughout the brain [3]. Additional neurophysiological roles for BDNF have been identified, including the modulation of neural activity, synaptic transmission, and plasticity [4] with key roles in cognition [5], sensory function [6,7], motor learning [8], and memory performance [9,10]. The expression level of BDNF is, at least partly, activity-dependent, as observed in the human cerebral cortex [11] as well as murine models, such that enriched environmental experiences and sensory stimulation increase BDNF levels in primary sensory cortices and hippocampus [12,13], while sensory deprivation leads to the opposite [14,15]. However, the synthesis and release of BDNF is heavily regulated by various physiological and pathological stimuli [3,16] and show significant genotype-expression interactions [17] and polymorphic variations [18]. In addition, altered central and peripheral plasma BDNF levels are significantly associated with numerous brain pathologies and, thus, have been described as biomarkers of a wide array of neuropsychiatric disorders [19,20]. Although the association between reduced BDNF levels and CNS pathologies is not necessarily causal, reduced BDNF levels are linked to increased neuronal impairment and degeneration, as observed in Parkinson’s disease patients [21]. Furthermore, reduced levels or hindered transmission of BDNF are expected to alter its corresponding cognitive and behavioral functions. Augmenting BDNF transmission, therefore, has been extensively investigated as a promising therapeutic approach in multiple brain pathologies. In this review, we describe the neurophysiology of BDNF transmission and receptor signaling and discuss the molecular and cellular mechanisms underlying its therapeutic potentials in neuropsychiatric disorders.

## 2. BDNF Transmission

**Figure 1:**
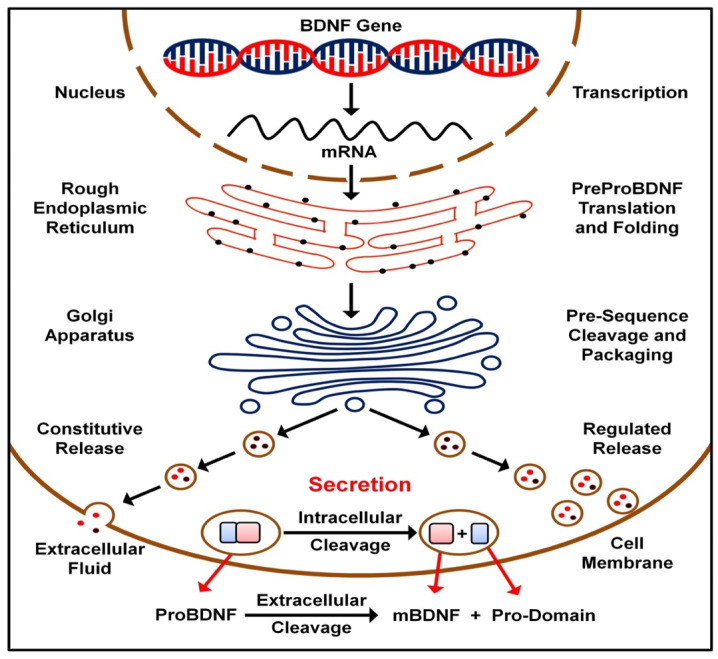
BDNF synthesis and release.

In humans, the proBDNF protein is encoded by the BDNF gene, which includes 11 exons and 9 promoters, with tissue and brain region-specific functionality producing alternatively spliced transcripts and regulated by various non-coding anti-sense ribonucleic acids (RNAs) from the anti-BDNF or *BDNFOS* gene [22]. Subsequent to translation, folding, and pre-sequence cleavage, the resultant proBDNF becomes packaged into vesicles for either constitutive (spontaneous) or regulated release [23]. The 32 kDa proBDNF can be cleaved by intracellular and extracellular proteases (e.g., plasmin and matrix metalloproteinases) to produce the mature ~14 kDa BDNF (mBDNF) and the propeptide proteins. When intracellularly cleaved, mBDNF and the propeptide can also be stored in dense core vesicles located in excitatory neuronal presynaptic terminals [24]. Therefore, neurons can release both the mature BDNF, as a primary trophic signal or neuromodulator, and the precursor proBDNF forms from presynaptic terminals (Figure 1). However, BDNF can also be released in a retrograde manner from postsynaptic cells to alter presynaptic activity [25] and mediate other specific functions such as synapse elimination in the developing cerebellum [26]. The mature form of BDNF signals primarily via the tropomyosin receptor kinase B (TrkB) receptor, while proBDNF binds to and activates the sortilin and p75 neurotrophin receptor (p75^NTR^), the latter of which is mainly expressed during development with lesser but maintained levels during adulthood [27]. Contrasting to the TrkB-mediated effects of mBDNF, the activation of sortilin and p75^NTR^, which is localized in dendritic spines and axon terminals by proBDNF, promotes cell death and attenuation of synaptic transmission through long-term depression (LTD), in addition to increasing anxiety and depression-like behaviors [28,29,30,31]. On the other hand, mBDNF actions mainly involve enhanced neuronal survival, growth, and synaptic activity [32]. However, mBDNF actions mediated via the TrkB receptor are highly diverse and can vary based on multiple factors. The multitude of TrkB-mediated functions is dependent on the specific BDNF mRNA species, pre- versus postsynaptic release, and receptor location, and intrinsic cell-specific interactors with the TrkB receptor [33]. Additionally, differential molecular alterations and cellular functions, such as synaptic activity and plasticity, are observed between acute and gradual activation of the TrkB receptor, and thus TrkB downstream signaling [34]. Similarly, transient activation promotes dendritic growth and spine morphogenesis, while sustained activation facilitates dendritic arborization and spinogenesis [35]. Another source of regulation is the activity-dependent modulation of TrkB receptor trafficking and cell-surface expression and translocation depending on the cell- and potentially synapse-specific degree of activity and BDNF release [36]. Furthermore, glial cells including astrocytes, which regulate BDNF recycling, oligodendrocytes, and microglia are also important targets and sources of BDNF, leading to yet another order of regulation and activity mediation [37,38,39,40]. Lastly, various TrkB receptor isoforms have been identified and mediate different functions, as discussed below. These findings indicate highly and tightly regulated control of endogenous BDNF synthesis, release, and transmission, allowing adequate direction of its diverse functions.

## 3. TrkB Receptor Signaling and Cellular Functions

### 3.1. TrkB Receptor Isoforms

The primary target of mBDNF, or simply BDNF, is the TrkB receptor, which belongs to the Trk family of tyrosine-kinase membrane receptors that mediate neurotrophins’ actions [41]. BDNF, as well as neurotrophin-4/5 peptides, bind the TrkB receptor with high affinity. It is encoded by the human *TrkB* gene and, under the influence of various promoters and splicing sites, transcribed into three main TrkB receptor splice variants. These include the primary full-length isoform (TrkB-FL), a truncated alternative isoform that lacks the intracellular tyrosine-kinase domain (TrkB-T1), and is hence unable to produce the fast downstream cytoplasmic signals, and another truncated isoform (TrkB-T2 or TrkB-T-Shc), also lacking the catalytic domain but exhibiting an additional Shc binding site [42]. However, evidence indicates that the truncated isoforms can regulate the activity of intracellular kinases [43,44]. Accordingly, differential effects are observed for the activation of TrkB receptor variants [45].

### 3.2. Truncated TrkB Receptor 

The TrkB-T1 isoform exhibits a dominant-negative functionality to inhibit TrkB-FL signaling in neurons and sequester BDNF; however, it is also responsible for other functions such as the regulation of cytoskeletal rearrangement [46]. The loci of expression between the splice variants also differ, and in glial cells TrkB-T1 has more than 100-fold higher expression than TrkB-FL. Additionally, it is found to induce inositol-1,4,5-trisphosphate (IP3)-mediated intracellular calcium signals in astrocytes, indicating key roles in neuroglial communication [47], and mediates astrocyte morphogenesis essential for downstream astrocytic support of neurons and synaptic function [48] as well as energy homeostasis and regulation of glutamate clearance [49]. During gliogenesis, BDNF-induced activation of truncated TrkB stimulates G-protein and protein kinase C (PKC) activation in neural stem cells to form glial progenitors and astrocytes [44]. The TrkB-T1 receptor can also alter cell signaling via Rho protein regulation. Using glioma cells, TrkB-T1 activation is found to dissociate the Rho guanine nucleotide dissociation inhibitor (Rho GDI1), thereby reducing the activity of RhoA, Rho-associated kinase (ROCK), p21-activated kinase (PAK), and extracellular-signal regulated kinase-1/2 (ERK1/2) [50]. Regulation of Rho proteins via TrkB-T1 is also found to inhibit glycine reuptake by astrocytes through endocytosis of glycine transporter [51]. On the other hand, little is known about the signaling mechanisms of TrkB-T1 in neurons, and its knockout mice have normal development in addition to hippocampal morphology, memory, basal transmission, and long-term potentiation (LTP). However, these mice showed increased anxiety and abnormal neurite length and complexity in basolateral amygdala neurons [52]. Very little is known regarding neuronal TrkB-T2 receptor signaling and functions; however, it shares similar properties with TrkB-T1, including the kinetics of signaling [43] and the inhibitory effect on TrkB-FL activation [53]. Nonetheless, these findings indicate that truncated TrkB isoforms are functionally independent receptors that alter cell signaling and balance BDNF-induced cellular functions. In fact, the imbalance between TrkB-FL and truncated TrkB-T1 activity is observed in various models of CNS injury and neuropathic pain, especially via TrkB-T1 upregulation [54].

### 3.3. Full-Length TrkB Receptor 

The predominant receptor isoform mediating the neurotrophic pro-survival effects, calcium signaling, and excitatory and inhibitory balance of BDNF in neurons is the non-truncated full-length TrkB-FL receptor exhibiting the tyrosine kinase catalytic subunit [55,56,57]. The binding of BDNF to the TrkB-FL receptor results in receptor phosphorylation, which leads to recruitment of intermediary proteins to bind various docking sites such as Shc adapter protein and activation of phospholipase C (PLC). Three main signaling cascades are subsequently triggered: the Phosphoinositide 3-kinase/Protein kinase B (PI3K/PKB-Akt) pathway, the mitogen activated protein kinase (MAPK) pathway, which is also known as the Ras-Raf-MAPK/ERK kinase (MEK)-ERK pathway, and the PLC pathway, which involves PLC-IP3-Ca^2+^ and PLC-diacylglycerol (DAG)-PKC signaling [58,59].

#### 3.3.1. The PI3K/Akt Pathway

The PI3K/Akt pathway interacts with the mammalian target of rapamycin (mTOR) and other downstream effectors and promotes protein synthesis, growth, proliferation, and survival [60]. Its upregulation, and subsequent BDNF-dependent activation, in neurons is supported to underlie the attenuation of ischemia-induced apoptosis by quercetin [61] and hippocampal neuronal injury in stroke via heme oxygenase 1 [62] as well as the cognitive enhancing action of L-3-n-butylphthalide in Alzheimer’s mice [63], anti-parkinsonian neuroprotection of curcumin [64], improved recovery from traumatic brain injury (TBI) by simvastatin [65], alleviation of oxidative glutamate toxicity by huperzine A [66], and the antidepressant action of liquiritigenin [67]. Additionally, activating the PI3K/Akt pathway via BDNF leads to increased dendritic translocation of postsynaptic density-95 (PSD-95) protein following N-methyl D-aspartate (NMDA) receptor activation, thus implicating this pathway in activity-dependent synaptic potentiation [68]. Indeed, BDNF-mediated PI3K/Akt signaling prevents the downregulation of synaptic plasticity-associated proteins in hippocampal neurons induced via hyperglycemia [69]. This is consistent with the loss of spine density and impaired synaptic plasticity and cognition concurrently with reduced BDNF levels due to insulin resistance [70], while improved insulin signaling upregulates BDNF, increases Akt phosphorylation, enhances cognition, and halts neuroinflammation and oxidative stress in Alzheimer’s disease [71,72]. Under physiological conditions, the BDNF-mediated activation of the PI3K/Akt/mTOR pathway controls the size of neuronal soma and dendrites; however, concomitant and coordinated activation of the MAPK pathway was found necessary to increase dendritic complexity [73]. These two pathways, both recruited via neurotrophins including BDNF, show bidirectional interactions to promote or inhibit one another, allowing further signal processing refinement [74].

#### 3.3.2. The MAPK Pathway

Upon TrkB stimulation, a cascade of kinases, namely Ras-Raf-MEK-ERK, become activated and trigger the second major signaling pathway. This MAPK pathway is an essential controller of the cell cycle [75], and similar to the PI3K/Akt pathway mediates, particularly via ERK1/2, various anti-apoptotic neuronal processes, both cytoplasmic and transcriptional, following BDNF stimulation [76]. Certain other MAPK subtypes mainly produce pro-apoptotic effects such as C-Jun N-terminal kinase (JNK) and p38-MAPK; however, the different MAPKs show complex interactions and exhibit the ability to promote opposing cellular actions depending on the type, intensity, and duration of cellular stimuli [77]. Apart from neuroprotection, MAPKs play key roles in a wide range of neuronal cellular processes in response to BDNF. These include anterograde dendrite-to-nucleus signaling and induction of immediate early gene expression [78], cyclic adenosine monophosphate (cAMP) response element-binding protein (CREB)-dependent LTP induction and dendritic Arc synthesis-dependent maintenance of LTP [79], and synapsin-dependent axonal growth [80] and presynaptic neurotransmitter release [81]. Additionally, BDNF-mediated MAPK signaling induces α-amino-3-hyroxy-5-methyl-4-isoxazole-propionic acid (AMPA) receptor trafficking and synaptic delivery [82]. As shown in the nucleus accumbens, however, BDNF effect on AMPA receptor surface expression is bidirectional as it can promote its synaptic delivery, and thus potentiation through ERK, or down-scaling following acute and sustained activation, respectively [83]. Accordingly, various observations support key roles for BDNF in glutamatergic plasticity in the nucleus accumbens and addiction-related activity modulation [84]. The above findings reveal a wide range of cellular actions, both fast cytoplasmic and slow transcriptional, mediated by BDNF-induced MAPK pathway signaling.

#### 3.3.3. The PLC Pathway

The third main signaling pathway of TrkB is triggered through PLC activation, especially PLCγ, which cleaves phosphatidylinositol 4,5-bisphosphate (PIP2) into the second messengers DAG and IP3. The latter activates IP3 channel receptors in the endoplasmic reticulum membrane, causing the release of stored Ca^2+^ into the cytosol [85]. Elevation of neuronal cytosolic Ca^2+^ concentration leads to a variety of downstream actions, particularly via calmodulin. These include the opening or enhanced permeability of various types of channels such as the membrane transient receptor potential-C (TRPC) cation channels in a store-operated mechanism [86], ligand-gated membrane channels such as NMDA receptors [87], and Ca^2+^-activated K+ channels, leading to long-lasting currents [88]. On the other hand, PLC-dependent but Ca^2+^-independent BDNF signaling also modulates Kv7/KCNQ potassium channels, as observed in parvalbumin-positive interneurons [89]. Additional actions include the Ca^2+^-dependent exocytotic [90], non-exocytotic [91], and reverse transport [92] neurotransmitter release, and activation of protein kinases including Ca^2+^/calmodulin-dependent protein kinases (CAMKs), which have key roles in BDNF-induced synaptic plasticity [93,94] and alteration of gene transcription via CREB [95]. The other messenger recruited via PLC activation is DAG, which mainly signals through PKC activation. This pathway is implicated in BDNF-mediated neuronal differentiation [96], survival [97], neurite outgrowth of dopaminergic neuron [98], NMDA receptor phosphorylation [99], AMPA receptor phosphorylation [100], γ-aminobutyric acid (GABA) receptor transcription [101], GABA-A receptor internalization during memory consolidation [102], spinal motor potentiation [103], and hippocampal activity integration and plasticity facilitation [104].

#### 3.3.4. Rapid Modulation of Ion Channels

BDNF actions mediated through the above signaling cascades occur mainly over periods of minutes to hours; however, BDNF is also observed to alter neuronal excitability within the second and even millisecond timescales, causing strong depolarization and trains of action potentials at nanomolar concentrations [105]. This rapid neurotransmitter-like BDNF signaling is mediated via direct modulation of membrane channels, especially voltage-gated ion channels [106]. This is observed for tyrosine kinase-dependent BDNF-induced excitation via NaV1.9 sodium channels [107] and calcium transients in dendritic spines via voltage-gated calcium channels, leading to LTP induction [108]. On the other hand, BDNF also modulates NaV1.2 channels through fast inactivation, resulting in reduced inward currents, thereby reducing excitability [109].

**Figure 2:**
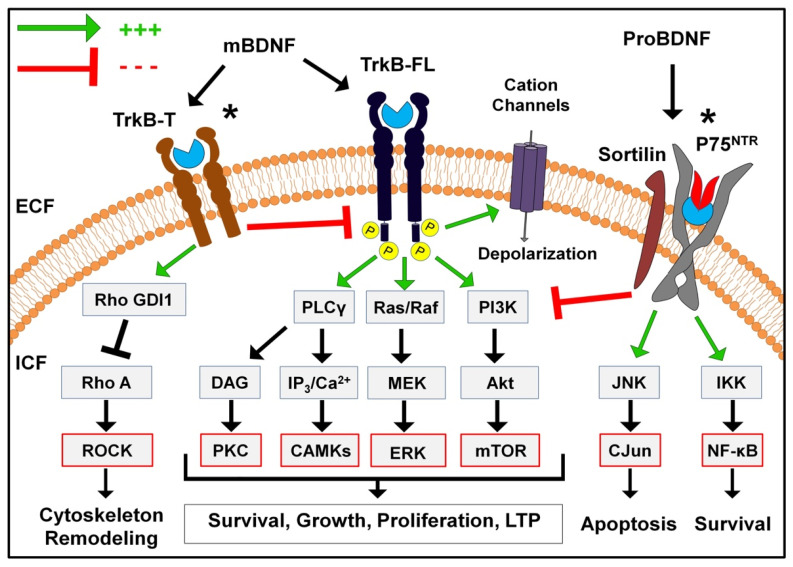
BDNF receptor signaling pathways. (*) different signaling pathways and cellular functions are triggered with heterodimer forms.

The above findings on TrkB-mediated signaling indicate that BDNF essentially affects all aspects of neuronal function, including the cell cycle, synaptic structure, neurotransmitter release, excitability, and plasticity. The diversity of TrkB-mediated cellular processes through its multiple signaling pathways (Figure 2) cross-talk with other cellular stimuli, directly interacting effectors and its bidirectional control nature, and therefore highlight the importance of balanced and coordinated spatiotemporal BDNF release in the maintenance of neuronal homeostasis. Insufficient or imbalanced BDNF transmission can thus have detrimental consequences on neuronal viability and function under certain pathological conditions. Accordingly, restoring or augmenting BDNF transmission and TrkB signaling, ideally in a differential and targeted manner, has emerged as a promising therapeutic approach in the potential management of various neuropsychiatric disorders through the engagement of multiple mechanisms.

## 4. Therapeutic BDNF Mechanisms

### 4.1. Neuronal Protection and Survival

BDNF is essential for neuronal survival under physiological conditions, and its mutation or deficiency lead to cell death that is more overt in specific neuronal populations [110,111]. In addition, exogenous supply of BDNF to neuronal cultures promotes survival by preventing apoptosis [112]. These pro-survival roles of BDNF can be through direct anti-apoptotic actions and/or via indirect protection from neuronal injury. Accordingly, BDNF-mediated signaling is able to block hypoxia-induced caspase-3 activation [113] and halt the upregulation of various apoptotic proteins, including phosphorylated C-Jun and cytochrome C due to cortical ablation [114]. The inhibition of p53 tumor suppressor protein and its upregulated modulator of apoptosis (PUMA) is also implicated in BDNF anti-apoptotic action [115]. Additionally, BDNF pre-treatment in neuronal cultures prevents excitotoxicity-induced apoptotic morphology and caspase activity through PI3K and MAPK pathways while increasing B-cell lymphoma 2 (Bcl-2) protein levels [116]. Although BDNF-mediated upregulation of NMDA receptor increases its calcium response and neuronal vulnerability to glutamate-induced necrosis, BDNF is still protected against excitotoxic apoptosis [117]. Furthermore, BDNF blocks caspase-3-independent cell death [118], potentially through the inhibition of apoptosis-inducing factor (AIF) mitochondrio-nuclear translocation, as observed in retinal photoreceptors [119]. Apart from ablative lesions, ischemia, and excitotoxicity, BDNF also exerts protective effects against metabolic stress, such as glucose deprivation-induced apoptotic cell death [120]. Another form of neuroprotection by BDNF involves its anti-oxidative effects, which attenuate neuronal injury under pathological conditions involving oxidative stress. Indeed, BDNF treatment or incubation can lead to reduction in markers of oxidative stress and upregulation of anti-oxidant enzymes such as glutathione reductase, glutathione peroxidase, and superoxide dismutase [121,122,123]. Lastly, BDNF exerts inhibitory effects on autophagy due to mitochondrial dysfunction [124], which is implicated in the multifaceted protective roles of BDNF in neurodegenerative disorders [125].

### 4.2. Synaptic Maintenence

The synaptic repair therapeutic strategy takes advantage of the dynamic nature of synaptic structure and function, which is the regenerative ability and reversibility of malfunction, and encompasses three main synaptic aspects: transmission, growth, and plasticity [126]. Accordingly, various neurodegenerative disorders are characterized by synaptic loss, which associates with sensory, motor, and cognitive impairments [127]. It is established that BDNF, generally, promotes both excitatory and inhibitory synaptic transmission via different mechanisms [128], induces dendritic growth and branching [129,130], increases synaptic number and density [131], and alters spine morphology and motility [132]. In addition, evidence indicates that BDNF-mediated synaptic modulation is bidirectional and regulates resting synaptic strength and functional plasticity within useful or optimal limits [133,134]. Accordingly, indirect evidence supports targeting BDNF transmission to enhance PKC activation for the prevention of synaptic loss in Alzheimer’s disease [135]. Indeed, more recent findings show that upregulated astrocytic BDNF production, conditioned to astrogliosis, improves cognitive deficits and recovers spine density, morphology, and markers such as PSD-95 in Alzheimer’s mice [136]. Various TrkB activating molecules have also been shown to prevent synaptic loss, such as 7,8-dihydroxyflavone (DHF) [137]. Therefore, the restoration and maintenance of synaptic activity, density, and structure represent a potentially disease-modifying therapeutic strategy in neurodegeneration.

### 4.3. Immunomodulation

Neuroinflammation is often described as a common pathological feature of multiple neurological and psychiatric disorders. While neuroinflammation is indeed central to some disorders, such as stroke, TBI, and infection, the involvement or alteration of certain immunological processes or mediators, which may not necessarily indicate inflammation, is a more accurate description in many others [138]. Accordingly, microglial activation or elevations in common indicators of an immune component, such as cytokines, are observed in a spectrum of CNS disorders, including many neurodegenerative diseases, epilepsy, depression, bipolar disorder, autism spectrum disorder (ASD), and schizophrenia [139,140,141]. BDNF-induced effects on microglial activity, astrocyte reactivity, and CNS cytokines within the context of halting disease progression or pathology will, therefore, be referred to as the immunomodulation mechanism. It should also be stressed that certain immune reactions or mediators may have damaging effects in one disorder while providing beneficial outcomes such as healing and repair in others [138]. Regarding the roles of BDNF and TrkB signaling, opposing actions are observed using different preparations and under different conditions. In microglial cultures, for instance, BDNF exerts activating effects while its sequestration inhibits microglial activation, motility, and production of tumor necrosis factor-α (TNF-α) [142]. Additionally, BDNF promotes astrocytic and microglial activation in the spinal dorsal horn of rats exhibiting cystitis-induced allodynia and upregulates various inflammatory markers such as TNF-α and interleukin-1β (IL-1β), while the opposite is observed upon TrkB blockade [143]. On the other hand, knockdown or pharmacological blockade of hippocampal BDNF increases microglial density, motility, surveillance area, and engulfment of synapses, as observed through in vivo and in vitro experimentation [144]. While many previous studies focused on the alterations of BDNF levels in CNS disorders in regards to their immunological or inflammatory components [145], definitive causal relations cannot be concluded. Nonetheless, recent studies directly investigated the immunomodulatory roles of BDNF showing “anti-inflammatory” actions counteracting brain “neuroinflammation” using various models. Indeed, the overexpression of BDNF blocks hyperglycemia-induced microglial activation and prevents the elevations in TNF-α, IL-1β, and nuclear factor-κB (NF-κB) [146], thereby reducing the central neuroinflammatory components associated with diabetic memory and cognitive impairments [147,148]. Additionally, BDNF supplementation prevents aging-related microglial sensitization and lipopolysaccharide (LPS)-induced dopaminergic neuronal loss and microglial activation, including morphological alterations; proinflammatory cytokine production; and p38-MAPK, JNK, and NF-κB signaling [149]. Similar immunoinhibitory effects attenuating astrocytosis, microcytosis, and cytokine production are observed via BDNF supplementation, together with fibroblast growth factor-2 (FGF-2) in a model of status epilepticus, leading to a significant reduction in seizure frequency [150]. Further supporting evidence for the therapeutic BDNF/TrkB immunomodulation mechanism is observed in models of post-stroke injury [151], depression [152], multiple sclerosis [153], and TBI [154] through anti-inflammatory effects that would protect from the potential damaging consequences of neuroinflammation or signaling disruptions by its mediators [155].

### 4.4. Plasticity Facilitation 

Synaptic plasticity, the activity-dependent modulation of synaptic transmission, encodes environmental experiences, thereby mediating various brain functions including development, learning, and memory, which ultimately shape cognition and behavior [156]. The induction of synaptic plasticity is governed by various rules and signals [157]. Among these is neuromodulatory input, which influences transmission to gate behavior-dependent plasticity induction and thus learning and memory [158]. BDNF acting as a neuromodulator facilitates synaptic plasticity via multiple mechanisms including the modulation of calcium dynamics, which regulate AMPAR trafficking [159] and retrograde signaling [160], attenuation of synaptic fatigue [161], excitability regulation of induction threshold [162], suppression of inhibitory transmission [163], neuronal synchronization by diminishing spike time jitter [164], functional synaptic clustering [165], and suppression of autophagy [166]. In addition, BDNF signaling serves in the synaptic tagging and capture of plasticity-related products [167] and maintenance of LTP [168,169]. Various studies have investigated the effects of reduced BDNF availability on synaptic plasticity using a mouse model (BDNF^Met/Met^ mice) that recapitulates the phenotype of a common human BDNF polymorphism (Val66Met), which leads to reduced activity-dependent BDNF release [170]. Indeed, BDNF^Met/Met^ mice exhibit synaptic plasticity impairments in the hippocampus [171], prefrontal cortex [172], amygdala [173], and striatum [174], thus implicating BDNF deficits in affective and cognitive aspects of neuropsychiatric disorders [175]. On the other hand, augmenting BDNF transmission rescues plasticity impairments in certain brain regions in murine models of Alzheimer’s disease [176], Huntington’s disease [177], fragile X syndrome [178], chronic intermittent hypoxia [179], schizophrenia [180], and aging [181]. As synaptic plasticity is impaired, or otherwise maladaptive, in various neuropsychiatric disorders [182,183] including depression [184], its facilitation by BDNF represents an important potential therapeutic mechanism. However, BDNF-induced spinal LTP of C-fiber synapses [185] can lead to hyperalgesia [186] via central sensitization, which, if it becomes maladaptive, can result in or promote chronic pain [187]. This is not restricted to the spinal dorsal horn as BDNF transmission is also implicated in orofacial neuropathic pain development and hypersensitivity, the treatment of which is challenging with few effective pharmacological therapeutic options [188,189,190,191]. These findings highlight the importance of selective regional targeting of BDNF transmission and TrkB signaling.

### 4.5. Secondary Neuromodulation 

Although BDNF itself exerts neuromodulatory effects on glutamatergic and GABAergic transmission, it also interacts with and affects other neuromodulators. Therefore, the BDNF-induced transmission or signaling alterations of neuromodulators, especially serotonin and dopamine, will be referred to as the secondary neuromodulation mechanism. Early evidence showed that BDNF administration, via midbrain or intracerebroventricular infusion, increases monoamine transmission, particularly serotonin and to a lesser extent dopamine, in various brain regions [192]. In relation to dopamine, BDNF is found to increase dopamine turnover [193] and potentiate its release in the striatum [194] and hippocampus [195]. In addition, BDNF upregulates the D_3_ receptor, which triggers levodopa sensitization [196] and potentiates the responses to D_3_ agonists, leading to improvements in motor behavior and recovery of striatal innervation and dendritic spines [197]. Accordingly, augmenting BDNF is a promising therapeutic strategy in Parkinson’s disease, not only by protecting from dopaminergic neuronal degeneration but also via augmenting dopaminergic transmission, as supported by preclinical and clinical evidence [198]. The effects of BDNF on hippocampal serotonin transmission, on the other hand, are mainly mediated by alteration of serotonin reuptake transporter (SERT) activity such that acute single injection of BDNF in the hippocampus leads to higher serotonin reuptake, lower extracellular levels, lower KCl-evoked increase in serotonin, and diminished signal amplitudes triggered by infused serotonin [199]. BDNF downregulation using heterozygous mice leads to opposite effects on serotonin by reduced hippocampal SERT activity, and these effects were observed to be region-specific [200]. Chronic administration of BDNF in the rat dorsal raphe nucleus causes significantly less-regular firing pattern of serotonergic neurons, suggesting that increased serotonin turnover could produce behavioral changes, including antidepressant effects [201]. Indeed, accumulating evidence indicates significant associations between reduced BDNF levels and depression and strong BDNF-mediated anti-depressant actions [202]. Genetic studies revealed interactions between BDNF polymorphisms and serotonin transporter variants on psychiatric functioning [203]. In addition, genetic interactions between BDNF and catechol-o-methyl transferase (COMT), the enzyme responsible for dopamine and norepinephrine degradation, translate into cognitive and behavioral alterations [204] and impact schizophrenia symptoms [205]. Interestingly, mutant mice that lack activity-driven BDNF expression exhibit reduced mRNA levels of 5-hydroxy tryptamine 1b (5-HT1b), 5-HT2a, and 5-HT5b serotonin receptors as well as dopamine D_2_ receptor subtype and alpha-1a/1d adrenergic receptors but increased levels for dopamine D_4_ receptor subtype in the frontal cortex. These mice showed depression-like behavior, impaired response inhibition, and inflexible learning, supporting the idea that reduced BDNF may lead to depression and schizophrenia through monoaminergic transmission alterations [206]. BDNF is important for dopamine sensitivity and the expression of dopamine D_3_ receptor subtype [207], the mRNA of which is decreased in patients with schizophrenia or bipolar disorder and increases following treatment; however, higher D_3_ levels correlated with negative schizophrenic symptoms [208]. In addition, higher frontal D_2/3_ binding potential also significantly correlates with positive symptoms [209]. On the other hand, BDNF heterozygous mice exhibit elevated extracellular dopamine levels compared to control but also show impairments in electrically evoked release and uptake of dopamine, suggesting differential roles of BDNF on tonic vs. phasic dopaminergic transmission [210]. Although the relationship between BDNF and schizophrenia is complex [211], these findings suggest that the degree of BDNF transmission can be targeted to alter, and potentially restore, the balance of dopaminergic transmission in schizophrenia [212]. This also applies for the complex BDNF-serotonin interactions in the understanding and treatment of various psychopathologies [213], especially in mood disorders such as anxiety and depression [214]. Interestingly, stress-induced BDNF dysregulation is brain region-specific, as observed in post-traumatic stress disorder (PTSD) in which BDNF mRNA is differentially downregulated in certain brain regions while being upregulated in others [215,216]. This further supports the BDNF stress-sensitivity hypothesis that BDNF dysregulation, as with the Val66Met polymorphism, predicts vulnerability to stress [217]. Indeed, TrkB receptor blockade enhances defeat-induced avoidance and PTSD-like symptoms in models of acute social and single-prolonged stress, which were mitigated by TrkB activation [218,219]. It should be stressed that secondary modulation of dopaminergic and serotonergic transmission by BDNF represents one mechanism affecting anxiety, depression, and schizophrenia as previously described mechanisms such as neuroinflammation, oxidative stress, and plasticity impairments are also involved and modulated by BDNF. Furthermore, the secondary neuromodulation mediated by BDNF could also impact other disorders such as delirium, which is commonly observed in critically ill elderly patients, either drug-induced or following trauma, and characterized by imbalanced neurotransmission including increased dopaminergic activity and altered serotonergic transmission [220,221]. Accordingly, BDNF might be expected to worsen or precipitate delirium by potentiating dopamine activity; however, evidence shows that lower BDNF levels are associated with reduced delirium recovery [222] and higher risk of postoperative delirium occurrence [223]. Interestingly, evidence suggests that corticotropin-releasing hormone-mediated BDNF depletion and spine loss could underlie delirium-like syndrome following trauma [224]. These findings highlight the significant impact of BDNF, via euromodulateon and other mechanisms, on various neuropsychopathologies and associated cognitive and behavioral impairments.

### 4.6. Preservation of Neurovascular Unit Integrity 

**Figure 3:**
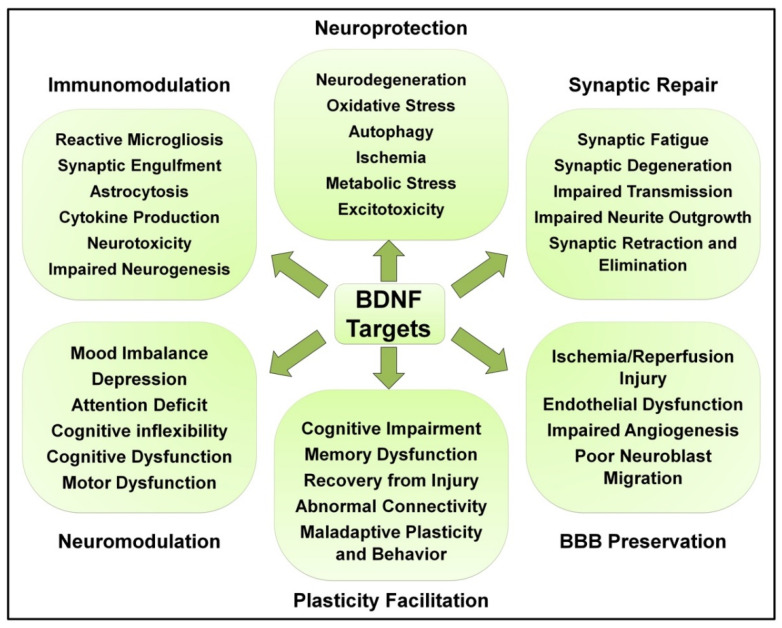
Summary of neuropsychiatric pathological alterations targeted by BDNF.

The integrity of the blood brain barrier (BBB) and neurovascular unit’s (NVU) cellular function is essential for CNS homeostasis via the regulation of influx/efflux transport and neurovascular coupling, ensuring adequate cerebral perfusion, while providing a protective barrier against potentially harmful molecules in the peripheral circulation [225]. Various pathological stimuli can lead to disruption or otherwise altered permeability and function of the BBB, such as ischemia, oxidative stress, and inflammation [226]. This is commonly observed in neurodegenerative disorders such as Alzheimer’s, Parkinson’s, and Huntington’s disease as well as multiple sclerosis, leading differentially to microbleeds, leakage, impaired transport function, cellular infiltration, and NVU cellular degeneration [227]. However, multiple other neuropsychiatric disorders have been shown to involve aspects of BBB disruption, including stress disorders such as depression and anxiety [228] as well as ASD [229], epilepsy [230], and schizophrenia [231]. As previously discussed, BDNF levels are reduced in the majority of aforementioned disorders, and its supplementation engages various converging therapeutic mechanisms to counteract associated pathologies that underlie BBB disruptions such as inflammation and oxidative stress. However, accumulating evidence indicates potentially direct roles for BDNF in the preservation of neurovascular integrity and function. The BDNF Val66Met polymorphism, for instance, is associated with poor angiogenic response following stroke through upregulated anti-angiogenic mediators [232]. In addition, BDNF is found to alleviate hyperglycemia-induced endothelial cell injury in the brain microvasculature [233], protect the integrity of the blood-spinal cord barrier following spinal cord injury [234], mediate cholic acid-induced protection of BBB integrity and NVU neuronal viability against hypoglycemic and ischemic injury [235], enhance vasculature-associated migration of neuroblasts towards ischemic lesions [236], facilitate BBB recovery from ischemia following release from astrocytes surrounding blood vessels [237], and trigger robust angiogenesis and promote brain endothelial cell survival [238]. These findings indicate that BDNF has direct protective effects on BBB integrity and NVU cell viability, representing yet another potential therapeutic mechanism in neuropsychiatric disorders. The various pathological processes potentially targeted by BDNF are summarized in Figure 3.

## 5. Neuropsychiatric Therapies Converge on BDNF: A Common Mediator

The therapeutic effects of BDNF essentially represent the target actions of various pharmacological agents used in the treatment of neurological and psychiatric illness. Many studies have investigated the effects of a wide range of medications on BDNF expression and its relation to associated therapeutic actions. In this regard, the focus was mainly directed towards certain anti-Alzheimer’s medications, antidepressants, and other miscellaneous synthetic drugs and natural compounds. Numerous pharmacological agents and herbal preparations have been identified and tested for the management of Alzheimer’s disease; however, only a few agents have been approved: the neuroprotective NMDAR antagonist memantine and the acetylcholinesterase inhibitors donepezil, rivastigmine, and galantamine [239,240]. In relation to memantine, it is found to upregulate BDNF expression [241], an effect supported to mediate memantine-induced enhancement of vascularization and recovery from stroke [242]; prevention of plasticity and memory impairment in a model of dopaminergic neurotoxin-induced Parkinson’s disease [243]; and antidepressant action counteracting chronic unpredictable stress-induced mitochondrial dysfunction, excitotoxicity, and oxidative stress [244]. Similarly, donepezil treatment causes a significant elevation of hippocampal BDNF expression in Alzheimer’s rats, mediating its cognitive enhancing action, attenuation of neurodegeneration, and restoration of synapse dendritic spines density [245,246,247]. The upregulation of BDNF is also observed for herbal preparations including gingko biloba, which leads to memory enhancement, prevention of neuronal apoptosis by lead poisoning, and neuroprotection against ischemic stroke [248,249,250,251]. In addition, various findings support the upregulation of BDNF as the mechanism underling the beneficial actions of many other botanical compounds in multiple neurological pathologies [252]. Furthermore, several lines of evidence indicate the upregulation of BDNF as a common transducer of anti-depressant action of many classical antidepressants and other agents such as ketamine [253]. Other medications shown to upregulate BDNF expression include second-generation antipsychotics such as olanzapine and clozapine [254], dopamine agonists such as rotigotine [255], the anesthetic dexmedetomidine, which enhances neuroprotection from cerebral ischemia/reperfusion injury [256,257], and delta opioid receptor agonists, which exert neuroprotective, anxiolytic, and antidepressant actions [258,259]. These findings support the notion that BDNF is potentially a common mediator of the therapeutic efficacy of centrally acting medications across an array of neuropsychiatric disorders. Although this is evident for few medications, definitive conclusions for most cannot be drawn based on associations. Therefore, further direct investigations are still required to evaluate whether attenuating drug-induced BDNF expression changes interfere with the intended therapeutic efficacy and if differences exist between agents of the same pharmacological class.

## 6. Current Status and Future Directions

Extensive research has been conducted to evaluate the therapeutic potential of BDNF in a wide range of brain pathologies, showing highly promising results. However, many limitations were recognized, especially regarding its delivery and central availability. Nonetheless, novel developments have been made to tackle this issue through various approaches such as gene therapy [260] and carrier-free stabilizing nanoencapsulation [261], which allows intranasal administration [262]. Other approaches include the use of TrkB receptor ligands such as DHF, small-molecule BDNF mimetics, and agonistic antibodies [263,264] as well as compounds boosting BDNF synthesis, transmission, and signaling, including natural products [126,265]. However, these direct and indirect approaches collectively referred to as BDNF-based therapies still face many other challenges [266]. Additionally, further research is still required to elucidate the network-specific functionality of BDNF, decipher how this modulates cognition and behavior, and uncover BDNF transmission disruptions in a disease-specific manner. Other considerations to take into account include systemic side effects, interactions with other disorders in which elevated BDNF levels can have harmful long-term consequences, and the potential impact of different polymorphisms on the efficacy of BDNF-based therapies.

To conclude, BDNF-mediated TrkB signaling controls a manifold of neuronal cellular functions and engages a multitude of converging and diverging molecular mechanisms that counteract multiple pathophysiological processes underpinning key aspects of neuropsychiatric disorders. These include (1) neuroprotection from apoptosis-inducing stimuli and stressors such as ischemia, excitotoxicity, energy imbalance, and oxidative stress; (2) synaptic regeneration and maintenance of activity and structure; (3) immunomodulation against microglial hyperactivity and abnormal production of inflammatory mediators; (4) facilitation and rescue of impaired and maladaptive synaptic plasticity; (5) secondary neuromodulation to alter dopaminergic and serotonergic transmission; and (6) preservation of BBB integrity and NVU cellular viability. Therefore, BDNF-based therapies carry significant therapeutic potentials in various neuropsychiatric disorders, but a set of challenges are still to be tackled.

## Data Availability

Not applicable.

## Abbreviations

5-HT: 5-hydroxy tryptamine; AIF: Apoptosis-inducing factor; AMPA: α-amino-3-hyroxy-5-methyl-4-isoxazole-propionic acid; ASD: Autism spectrum disorder; BBB: Blood brain barrier; Bcl-2: B-cell lymphoma 2; BDNF: Brain-derived neurotrophic factor; CAMK: Ca^2+^/calmodulin-dependent protein kinase; cAMP: Cyclic adenosine monophosphate; CNS: Central nervous system; COMT: Catechol-o-methyl transferase; CREB: cAMP response element-binding protein; DAG: Diacyl glycerol; DHF: 7,8-dihydroxyflavone; ERK: Extracellular-signal regulated kinase; FGF-2: Fibroblast growth factor-2; GABA: γ-aminobutyric acid; IL-1β: Interleukin-1β; IP3: Inositol-1,4,5-trisphosphate; JNK: C-Jun N-terminal kinase; LPS: Lipopolysaccharide; LTD: Long-term depression; LTP: Long-term potentiation; MAPK: Mitogen activated protein kinase; mBDNF: Mature BDNF; MEK: MAPK/ERK kinase; mTOR: Mammalian target of rapamycin; NF-κB: Nuclear factor-κB; NMDA: N-methyl D-aspartate; NVU: Neurovascular unit; P75^NTR^: p75 neurotrophin receptor; PAK: P21-activated kinase; PI3K: Phosphoinositide 3-kinase; PIP2: Phosphatidylinositol 4,5-bisphosphate; PKB/Akt: Protein kinase B; PKC: Protein kinase C; PLC: Phospholipase C; PTSD: Post-traumatic stress disorder; PSD-95: Postsynaptic density-95; PUMA: P53 upregulated modulator of apoptosis; Rho GDI1: Rho guanine nucleotide dissociation inhibitor; RNA: Ribonucleic acid; ROCK: Rho-associated kinase; SERT: Serotonin transporter; TBI: Traumatic brain injury; TNF-α: Tumor necrosis factor-α; TrkB: Tropomyosin receptor kinase B; TrkB-FL: Full-length TrkB receptor; TrkB-T: Truncated TrkB receptor; TRPC: Transient receptor potential-C.
